# B cell intrinsic expression of IFNλ receptor suppresses the acute humoral immune response to experimental blood-stage malaria

**DOI:** 10.1080/21505594.2020.1768329

**Published:** 2020-06-07

**Authors:** William O. Hahn, Marion Pepper, W. Conrad Liles

**Affiliations:** a Division of Allergy and Infectious Diseases, Department of Medicine, University of Washington, Seattle, USA; b Department of Immunology, University of Washington, Seattle, USA

**Keywords:** Malaria, plasmodium, type III interferon, interferon-λ, humoral immune response

## Abstract

Antibodies play a critical protective role in the host response to blood-stage malaria infection. The role of cytokines in shaping the antibody response to blood-stage malaria is unclear. Interferon lambda (IFNλ), a type III interferon, is a cytokine produced early during blood-stage malaria infection that has an unknown physiological role during malaria infection. We demonstrate that B cell-intrinsic IFNλ signals suppress the acute antibody response, acute plasmablast response, and impede acute parasite clearance during a primary blood-stage malaria infection. Our findings demonstrate a previously unappreciated role for B cell intrinsic IFNλ-signaling in the initiation of the humoral immune response in the host response to experimental malaria.

## Introduction

Malaria has the highest incidence, prevalence, morbidity, and mortality of any human parasitic infection [1]. *Plasmodium*-specific antibodies protect against clinical disease but are short-lived after natural infection, suggesting a defect in the memory phase of the humoral response [2–7]. During primary infection, the first antibodies to appear in plasma are generated by short-lived effector B cells (“plasmablasts”) [8]. In contrast, the memory phase of the humoral response is primarily driven by B cells that can be generated in a germinal center (GC) and survive to become either memory B cells or long-lived plasma cells [9–12]. In experimental systems, individual B cell clones with identical B cell receptors (BCR) can both enter into the memory compartment or form plasmablasts early after activation [11,13,14], suggesting that environmental cues extrinsic to the cell are a potential determinant for B cell fate decisions. Insight into the early factors that shape early B cell responses is important for understanding the basis for the poor humoral memory observed after blood-stage malaria infection, a critical obstacle for the development of an effective vaccine.

The cytokine environment where a naive B cell encounters its cognate antigen is important for the initial B cell response [15,16]. Interferons (IFNs) are among the first cytokines produced by the innate immune system in response to infection [17], are abundant during early blood-stage malaria [18], and are therefore logical candidates to influence early *Plasmodium-*specific B cell fate decisions. There are three families of IFNs: Type I (IFN⍺/β), Type II (IFNγ) and Type III IFN (IFNλ). Despite substantial overlap in the gene programs induced by all IFNs, IFN signaling occurs via three distinct family-specific receptors, and each IFN family can have different effects on the B cell response depending on the context of the immune stimulus [19]. For example, Type I IFN signals in B cells are critical for lymphocyte retention inside lymph nodes [20], development of alloantibodies to exogenous antigens on erythrocytes [21], and initiation of the humoral response during influenza infection [22]. In contrast, blocking Type I IFN signals has been demonstrated to improve humoral function in the context of chronic LCMV infection [23]. For blood-stage malaria infection, our group and others have determined that Type I IFN signals enhance parasite clearance [24–27] whereas other groups have had different results [28,29]. Similar to Type I IFN, Type II IFN can also have different effects on the humoral response depending on the biological context. In both human and murine *Plasmodium* infection, excess IFNγ signaling has been linked to poorly functional “atypical” memory B cells and reduced antibody formation [30–32]. Additionally, decreased IFNγ signaling is associated with fewer GCs and reduced antibody output in response to either alloantigens or autoantigens [33–35].

In comparison to Type I and Type II IFN, much less is known about how IFNλ (Type III IFN) influences *in vivo* humoral responses. IFNλ plays a critical in host protection against rotavirus infection in enterocytes and is important for limiting influenza replication in the respiratory epithelia, suggesting a critical role at barrier interfaces [36–38] . The role of IFNλ likely extends beyond the direct effects at mucosal surfaces, however, and likely has important implications for the humoral response. B cells express IFNλ receptor mRNA [39], IFNλ activates B cells *in vitro* [17,39], and exogenous IFNλ reduces antibody secretion during stimulation with influenza antigens [40]. The magnitude of long-term antibody titers following acute LCMV infection was not affected by IFNλ signals, however, but the role of IFNλ for the acute antibody response is unknown [41]. While IFNλ is one of the top five differentially regulated cytokines in the blood of patients with febrile malaria (as compared to non-febrile malaria) [18], the consequences of IFNλ signals for the host response to blood-stage malaria have not been previously investigated.

Understanding the interplay between IFNλ, blood-stage malaria, and the B cell response is important because polymorphisms in the human IFNλ locus are associated with the immune response to both infections and vaccinations. Strong evolutionary pressure is thought to have caused the striking regional segregation in the population genetics of IFNλ and genetic variation in the IFNλ locus largely explains the poor response to immunotherapy treatment for hepatitis C in patients of African descent [42–44]. While there is consensus that alleles more common in African populations are associated with lower expression of IFNλ, the evolutionary pressures driving this variation are unclear [40,45–47].

IFNλ signals via a specific receptor, the IFNλR which is formed when the the IFNλR1 subunit combines with the beta subunit of the IL-10 receptor to form a functional heterodimer [48]. Mice with a targeted ablation of the IFNλR1 (*Ifnlr1^−/-^*) are therefore incapable of responding to IFNλ in a manner similar to mice with targeted disruption of all IFNλ cytokines (*Ifnl2^−/-^/Ifnl3^−/-^*) [37,49]. To explore the potential role that IFNλ plays in the humoral response to blood-stage malaria infection, we infected *Ifnlr1^−/-^* mice with *Plasmodium yoelii* as model non-lethal blood-stage malaria infection. We observed that the absence of IFNλ signaling decreased parasite burden, increased early antibody titers, and increased the number of malaria-specific plasmablasts. Furthermore, these responses depended upon B cell-intrinsic expression of IFNλR *in vivo*. Our data clearly show that IFNλ signals have strong influence on the acute B cell response during blood-stage malaria infection.

## Results

### Genetic deletion of IFNλ receptor reduces parasite burden during initial blood-stage malaria infection

**Figure 1.**
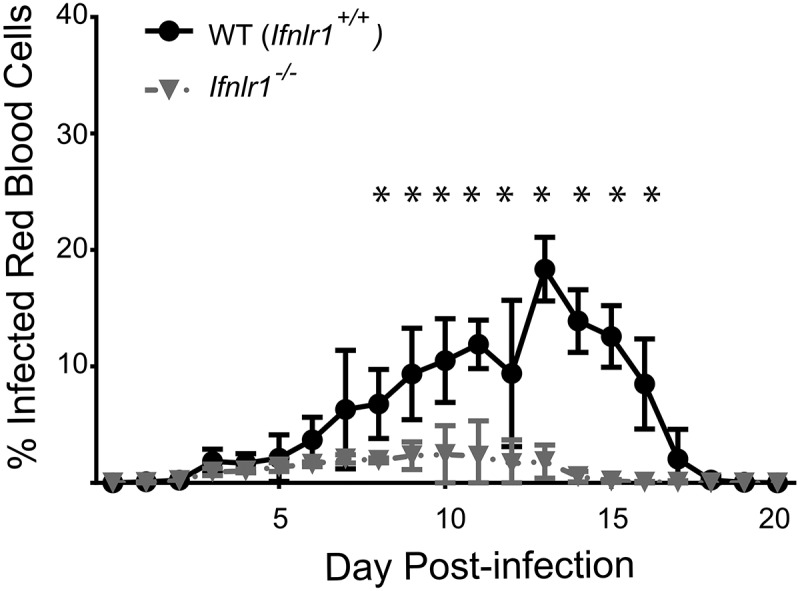
Absence of interferon lambda leads to improved parasite control during blood-stage malaria infection.

The biological role of IFNλ produced in response to *Plasmodium* infection is unknown. Whereas transcription of IFNλ mRNA increases substantially during acute stage blood-stage malaria infection [18], chronic malaria infection is associated with lower levels of plasma IFNλ [50]. We therefore sought to assess the biological role of IFNλ during blood-stage malaria infection *in vivo*. To test the effects of IFNλ on the outcome of blood-stage malaria, we infected mice with a global deficit in IFNλ signaling (*Ifnlr1^−/-^* mice) [51] with *Plasmodium yoelli 17XNL*, a non-lethal murine model of malaria. Given that both genetic background [52] and differences in microbiome [53] influence the course of murine malaria infection, all experiments were performed using sex-matched littermate controls born from *Ifnlr1^±^* by *Ifnlr1^±^* heterozygote pairings in order to minimize confounding variables. Using flow cytometry to measure the percentage of erythrocytes containing parasites (parasitemia) [24], we determined that parasitemia was strongly decreased in *Ifnlr1^−/-^* starting at day 10 post-infection when compared to littermate controls (Figure 1). Because control animals do not experience mortality or weight loss in this model [24], no differences were observed with respect to these clinical variables (data not shown). From these data, we concluded that genetic deletion of IFNλ signaling is associated with a substantial decrease in parasite burden during primary blood-stage malaria infection.

### Genetic deletion of the IFNλ receptor increases plasmablast formation and acute malaria-specific antibody production

**Figure 2.**
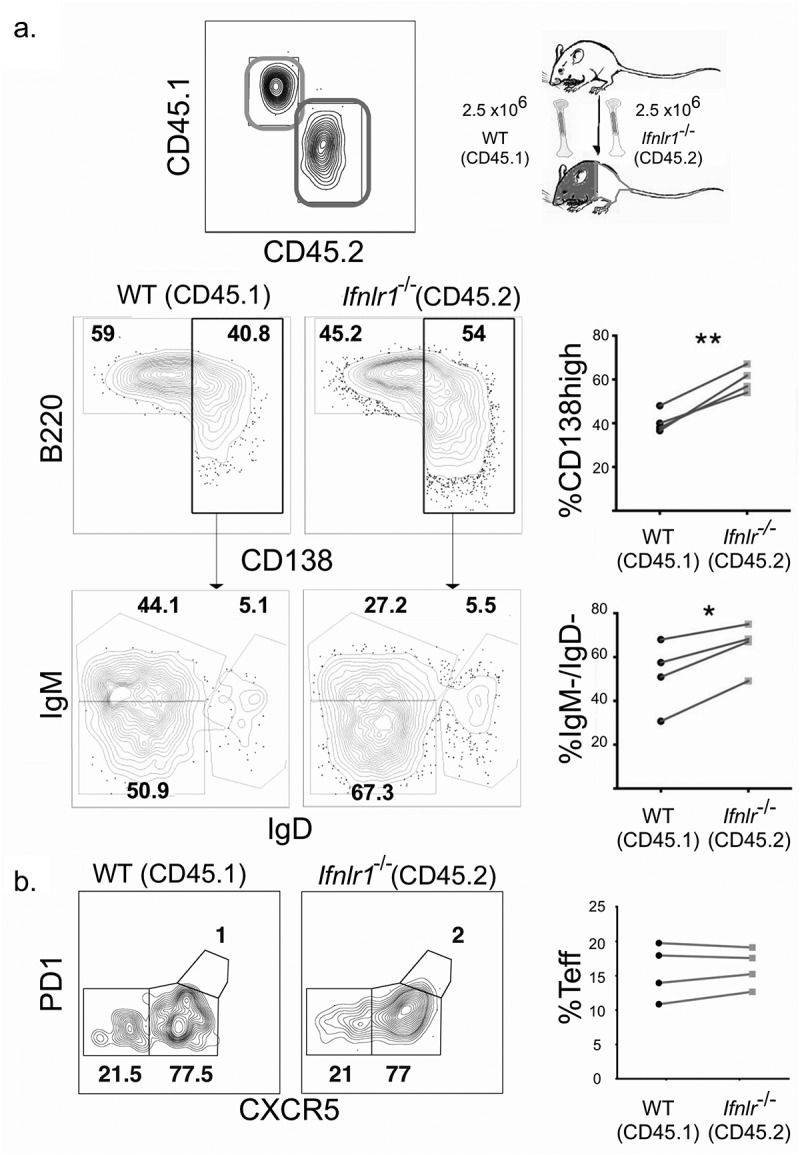
Absence of interferon lambda leads to increased antibody titers and increased plasmablast numbers.

The timing of reduction in parasite burden we observed (starting 10 days after infection) suggested a difference in the adaptive immune response. In the *P. yoelii 17XNL* model, T-and-B cell deficient mice (*RAG^−/-^* mice) first develop higher parasitemia compared to WT controls starting around days 8–10 post infection [54–56]; in contrast, control of parasite replication driven by the innate system appears earlier (approximately day 5) [54–56]. Antibodies are absolutely required for both parasite clearance and protection against reinfection in the *P. yoelli 17XNL* model [57]. We therefore hypothesized that differences in the humoral response driven by the lack of IFNλ signals could explain the observed difference in parasite control. To test this hypothesis, we measured antibody titers against a truncated carboxy terminus of the blood-stage antigen merozoite surface protein (MSP1) shown to be critical for infection by ELISA [24]. We decided to measure specifically the IgG_2_ _c_ because the IgG2 c antibody appears early in plasma and can confer protection in murine models of blood-stage malaria [58–60]. Furthermore, we decided to measure acute antibody titers immediately prior to divergence of parasite burden between *Ifnlr1^−/-^* mice and littermate controls, given that variations in inoculum and ongoing inflammation can have dramatic effects on antibody titers during infection with malaria [24] and other pathogens [61–63]. Titers of anti-MSP1 IgG_2_ _c_ and IgM were increased at day 7 post-infection in *Ifnlr1^−/-^* mice vs. littermate controls (Figure 2A). From these data, we concluded that *Ifnlr1^−/-^* mice had higher levels of antibody isotypes associated with protection when compared to littermate controls just prior to the divergence in parasite burden, demonstrating that antibody level did not reflect differences in antigen exposure.

Next, we determined whether there were differences in the B cell response that could potentially explain the difference in observed plasma antibody titers. The acute antibody response to infection is initiated with a subset of short-lived antibody secreting B cells called plasmablasts [15,64]. These cells are defined by surface expression of CD138+ (syndecan-1), and provide minimal contributions to the memory pool due to rapid cell death from apoptosis [8]. During a primary immune response, antibodies generated by plasmablasts are capable of directly neutralizing some infections [65]. As we had observed differences in plasma titers of antibodies in *Ifnlr1^−/-^* mice vs. littermate controls, we hypothesized that there would be differences in the early malaria-specific plasmablast response. To test this hypothesis, we utilized previously described B cell tetramers in combination with conventional flow cytometry [24]. Magnetic bead enrichment of B cells capable of binding a tetramer that incorporates the carboxy terminus of MSP1, enables the enumeration and characterization of the MSP1-specific B cells responding to infection without *ex vivo* manipulation [24,58]. We determined that the observed differences in antibody titers on day 7 post-infection were reflected in the number of MSP-specific plasmablasts, as *Ifnlr1^−/-^* mice had increased numbers and percentages of MSP-specific plasmablasts (Figure 2B). We also observed that the differences in plasma IgG2 c was also reflected in increased numbers and percentages of IgG negative/IgG2 c negative MSP-specific plasmablasts. From these data, we concluded IFNλ signaling suppresses the acute humoral response to blood-stage *Plasmodium* infection.

### Genetic deletion of IFNλ receptor shifts CD4 + T cell differentiation toward an effector phenotype

**Figure 3.**
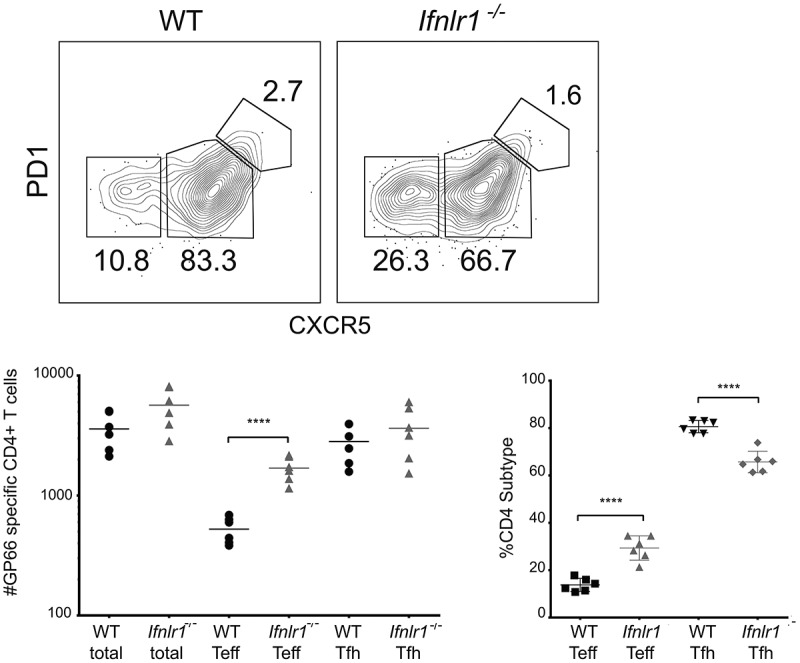
Absence of interferon lambda leads to increased CD4+ T effector cells.

Because CD4 + T cells are known to play a critical role in both the activation of B cell responses during a blood-stage *Plasmodium infection* [66,67], we hypothesized that IFNλ could also influence the CD4 + T cell response. IFNλ has been demonstrated to modulate CD4+ T cell differentiation in both *Ifnlr1^−/-^* mice [41] and humans given exogenous IFNλ[68]. To assess the role of IFNλ on the development and differentiation of CD4+ T cells, we used a transgenic *P. yoelii 17XNL* strain that stably expresses the LCMV epitope GP66^+^ [24]. This parasite allows for quantitation and phenotypic assessment of antigen-specific CD4+ T cell cells via flow cytometric analysis of CD4+ T cells that bind the fluorescently-conjugated GP_66_ I:A^b^ tetramer [69]. Although the total number of GP66+ CD4+ T cells were similar in *Ifnlr1^−/-^* mice and littermate controls on day 7 post-infection, there were substantial differences in the cellular phenotype of the antigen-specific CD4+ T cell response. Specifically, *Ifnlr1^−/-^* mice had a greater number and percentage of antigen-specific T effector (Teff) (defined as GP66+, CD44+, CXCR5^low^) [70] and fewer CD4+ T follicular helper (Tfh) cells (defined as GP66+, CD44+, CXCR5^high^) when compared to littermate controls (Figure 3). From these data, we concluded that the absence of IFNλ signals skews the CD4 + T cell response toward an effector response during the initial phase of the immune response to blood-stage *Plasmodium* infection prior to divergence in parasite burden.

### Absence of cell-intrinsic IFNλ signals favors plasmablast formation but does not affect CD4 + T cell differentiation

We had observed differences in the cellular differentiation of both CD4 + T cells and B cells, so we decided to investigate which effects, if any, were a result of direct IFNλ signals. Both direct and indirect cellular effects on lymphocytes could be plausible. IFNλ has been shown indirectly mediate the differences in CD4+ T cell response [41,71]. B cells are directly responsive to IFNλ in vitro [72]. Additionally, *in vivo* interactions between CD4+ T cells and B cells can also affect the differentiation of each cell type, suggesting that either B cells (or CD4+ T cells) could be driving the effector phenotype [15].

**Figure 4.**
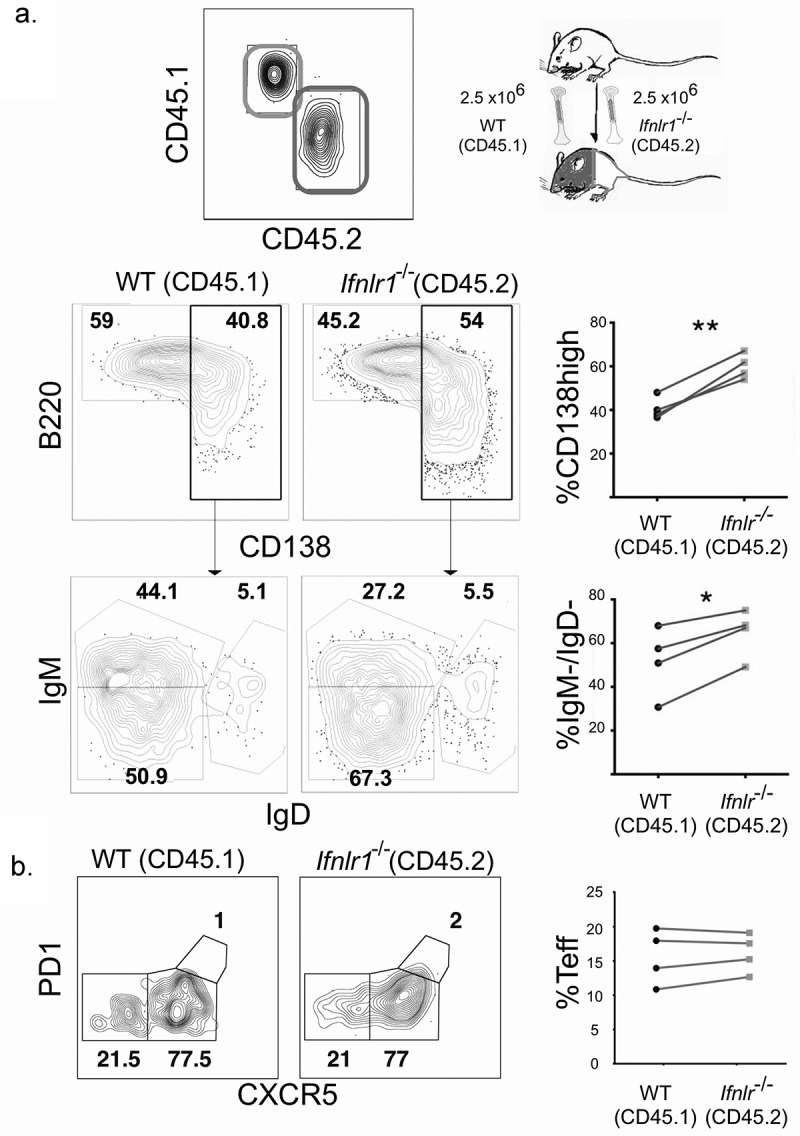
Interferon lambda signals suppress plasmablast formation in a B cell-intrinsic fashion.

We hypothesized that the effects of IFNλ for B cell differentiation were due to B cell intrinsic signals because IFNλ suppresses B cell proliferation and antibody secretion *in vitro* in PBMCs [40]. To test this hypothesis, we utilized a congenically-labeled mixed bone marrow chimera system in which cell intrinsic effects can be examined in the same mice. Lethally irradiated CD45.1/CD45.2 mice were reconstituted with bone marrow from both WT CD45.1 and *Ifnlr1^−/-^* CD45.2 mice. The resulting experimental system allowins for testing whether the effects of IFNλ are intrinsic to any hematopoietic cell of interest. Additionally, the system normalizes the cytokine environment, antigen load, host background, and cellular interactions. After allowing the mixed bone marrow chimera mice to reconstitute, we infected mice with non-lethal transgenic *P. yoelii 17XNL GP66* as before. We assessed the antigen-specific CD4+ T cell and B cell responses on day seven post-infection. We observed that the *Ifnlr1^−/-^* (CD45.2) B cells formed isotype-switched MSP1-specific plasmablasts at a higher frequency than WT (CD45.1) cells (Figure 4A). When plasmablasts were gated out from the total B cell population, no differences were observed in the formation of isotype-switched memory B cells or germinal center precursors (data not shown). We observed no effects on CD4+ T cell differentiation. From these data, we concluded that IFNλ signals acting directly upon B cells were responsible for the difference in plasmablast formation in response to blood-stage malaria infection. Consistent with other infectious models [41,71], we observed no differences in the antigen-specific CD4 + T cell response between WT (CD45.1) and *Ifnlr1^−/-^* (CD45.2) cells (Figure 4B), demonstrating the shift toward an effector response we observed in the CD4 + T cells of *Ifnlr1^−/-^* mice was due to indirect (cell-extrinsic) effects.

### IFNλ−mediated control of parasitemia and plasmablasts is due to B cell-intrinsic signals

**Figure 5.**
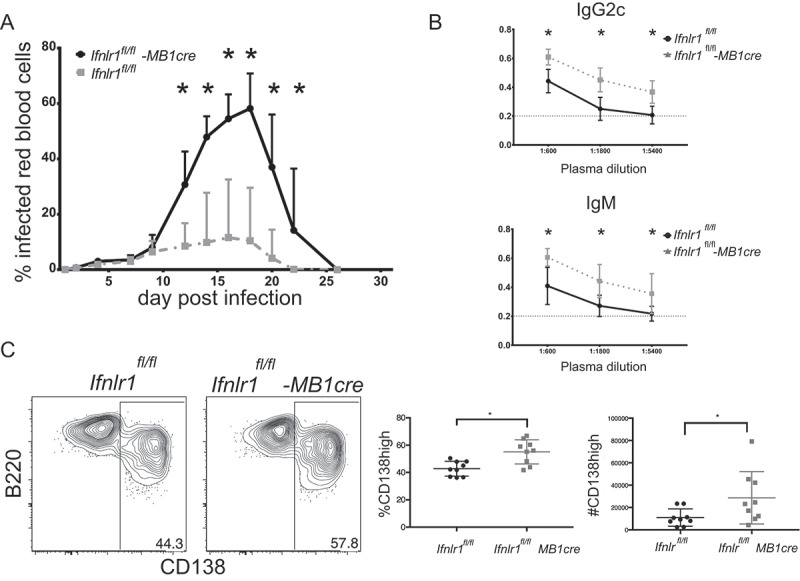
Improved control of blood-stage infection with absence of Interferon lambda-specific B cells.

Since we observed that absence of B cell-intrinsic IFNλ signaling increased plasmablast formation, we hypothesized these effects were also responsible for mediating the improved control of parasite burden in *Ifnlr1^−/-^* mice. However, CD4+ T cells have also been demonstrated to directly mediate protection against blood-stage *Plasmodium* [73]. To test whether the absence of IFNλ signals on B cells was directly responsible for improved parasite control, we generated a mouse that conditionally lacked IFNL1 R in the B cell compartment. Transgenic mice that express Cre under the B cell – specific MB1 promoter were crossed with mice with a floxed IFNλ receptor allele (MB1-cre x *Ifnlr^fl/fl^*) [74,75]. The resulting offspring therefore lack expression of the IFNλ receptor in the B cell compartment [75]. To test whether B cell-restricted IFNλ signaling recapitulated the results we see in the chimeric setting and were responsible for parasite control, we infected MB1-cre *Ifnlr1^fl/fl^* mice with *P. yoelli 17XL GP66* and measured daily parasitemia. Similar to our observations in mice with a global deficit in the IFNλ receptor, we observed improved control of parasitemia starting at day 10 in MB1-cre *Ifnlr1^fl/fl^* mice when compared to littermate controls that lack the cre allele (*Ifnlr1^fl/fl^*) (Figure 5A). When we assessed the MSP1-specific B cell response, we again determined that there were increased plasmablasts (Figure 5B) in cre-sufficient mice as compared to littermates who lack the cre-allele. Similar to Ifnlr1-/- mice, MB1-cre *fnlr1*^fl/fl^ mice had increased titers when compared to littermate controls. As expected, we observed no effects on the CD4 + T cell response (data not shown). These data indicate that IFNλ signals on B cells control parasite burden; moreover, consistent with the data from mixed bone-marrow chimeras (see Figure 4), the observed increased formation of plasmablasts was due to B-cell intrinsic IFNλ signals. We would note, however, that the parasite burden in the control *Ifnlr1^fl/fl^* mice was higher (~50% peak) than the burden in the original C57BL6/J-background control mice (*Ifnlr1^+/+^*) at peak (~20%), suggesting potential differences in experimental conditions or host genetic background.

## Discussion

Using a murine model of blood-stage malaria infection, we have determined that the absence of IFNλ improves early parasite control via direct effects on B cells. Our findings that IFNλ signals impede parasite clearance during non-lethal blood-stage infection with *P. yoelii* are reminiscent of the role of anti-inflammatory cytokines such as IL-10, as mice with disrupted IL-10 signaling have reduced parasite burdens during non-lethal blood-stage malaria infection [76]. Our findings that removal of IFNλ improves acute parasite control are consistent with its physiological role in other systems, as IFNλ has been shown to directly suppress neutrophil-mediated inflammation in models of drug induced colitis [77] and thrombosis [78]. The inferred “suppressive” effect during non-lethal blood-stage malaria (where removal improves the acute host response) is interesting given that the functional receptor for IFN-λ shares a common subunit with the IL-10 receptor [48] and the IL-10 family has been described as the prototypical anti-inflammatory cytokine [79].

Our findings that the *in vivo* effects of IFNλ signals repress plasmablast formation add to the understanding of the biological role of this cytokine during the humoral response to systemic pathogens. Previous *in vitro* investigations have reached differing conclusions regarding the biological effects of IFNλ signaling for B cells. Exogenous administration of IFNλ reduced both proliferation and activation of B cells during stimulation with influenza antigens [40] whereas in vitro administration of IFNλ in conjunction with TLR7 agonists enhanced Ab secretion and proliferation [39]. The discrepancies between whether IFNλ stimulates or suppresses the B cell effector functions are similar to the discrepancies of the biological role of both Type I and Type II IFN. We suspect that, like other IFNs, the *in vivo* role of IFNλ depends on the immune context. In general, our observation that IFNλ-deficient B cells form plasmablasts at a higher rate during blood-stage malaria infection are more in keeping with an “suppressive” role of IFNλ. While we did not formally assess proliferation, plasmablasts undergo rapid proliferation and are strongly associated with inflammatory disorders such as systemic lupus erythematosus [80]. An alternative, non-exclusive explanation could be that IFNλ induces plasmablast-specific cell death as was recently demonstrated in intestinal cells [81]. The exact mechanism by which IFNλ signals reduce the number of plasmablasts should remain an active area of investigation.

IFNλ signals appears to influence the CD4+ T cell response during blood-stage malaria in an indirect fashion. There is no consensus as to whether T cells can respond to IFNλ directly, as some groups have reported direct effects of exogenous IFNλ for CD4 + T cells (typically on *ex vivo* human T cells) [82,83] whereas other groups using both human or murine systems have not found direct effects [41,71,84,85]. Our mixed bone marrow chimera experiments demonstrate that the shift toward an effector response in *Ifnlr1^−/-^* mice during blood-stage infection is not mediated by direct IFNλ signals on CD4 + T cells, in keeping with observations using similar approaches [41,71]. Furthermore, our experiments in MB1-cre *Ifnlr1^fl/fl^* mice demonstrate that the CD4+ T cell effector bias we observed is not mediated by IFNλ signals on B cell. The cell type responsible for IFNλ-mediated alterations in the CD4+ T cell response during blood-stage malaria infection should be a focus of further investigations. Because conflicting evidence exists regarding the role of CD8 + T cells during experimental acute blood stage malaria [86–91], we did not investigate the role of the CD8 + T cell population in our model. As other groups have reported increased numbers of CD8+ T cells in *Ifnlr1^−/-^* mice during the acute response to LCMV, [41], there could be a potential role for alterations in the CD8+ T cell population in *Ifnlr1^−/-^* mice. Potential effects of IFNλ on the CD8+ T cell population should remain an active area of investigation for future studies. Similar to CD4+ T cells, CD8+ T cells do not respond directly to IFNλ signals, suggesting that any potential role would be indirect [41,71].

Our findings also add to the body of literature suggesting that the biological role of IFNλ is distinct from other IFNs. Forero et al. recently demonstrated that Type III IFNs preferentially elicit genetic programs associated with tissue repair when compared to Type I IFNs [92] Additionally, *Ifnlr1^−/-^* mice had different alterations in the immune response during intransal vaccination with attenuated influenza compared when compared Type I IFN receptor-deficient (*IFNAR1*^−/-^) mice [93,94].

Our findings demonstrating that IFNλ suppresses the acute B cell response to blood-stage malaria suggest that the biological role of IFNλ extends beyond the barrier interface. Our findings have potential implications for antibody-mediated autoimmune diseases where plasmablasts are thought to contribute to disease pathogenesis such as SLE or rheumatoid arthritis. Additionally, our findings suggest that IFNλ can modulate the acute humoral response. The effects of B-cell intrinsic IFNλ for the memory response should be an active area of future investigation.

## Materials and methods

### Study approval

All experiments involving animals were performed in accordance with the University of Washington Institutional Animal Care and Use Committee guidelines.

### Mice

Male 6-to-8 week old C57BL/6 J, SJL 45.1, and MB1^cre/cre^ mice were purchased from Jackson ImmunoResearch Laboratories and maintained under specific-pathogen free conditions per the University of Washington Guidelines. *Ifnlr1^−/-^* and *Ifnlr1^fl/fl^* mice were provided as a kind gift by Michael Gale Jr. *Ifnlr1^−/-^* mice were bred from heterozygotes pairings with genotyping as previously described [41]. All experiments were performed in accordance with University of Washington Institutional Care and Use Committee guidelines.

### Mixed bone marrow chimeras

Mixed bone marrow chimeras were generated as previously described [70]. Bone marrow cells were depleted of T and NK cells and C57BL/6 J SJL.1 (CD45.1) and *Ifnlr1^−/-^* cells were counted and mixed in equal proportions with 2.5 million cells of each type. Recipient mice were lethally irradiated with 1000 rads, and bone marrow was reconstituted via retroorbital injection of marrow cells. Mice were allowed to reconstitute at least eight weeks prior to infection. Representative flow cytometry gating scheme for identification of congenically marked, antigen-specific B cells on day seven post infection. Plots representative of four mice from two separate experiments are shown. Statistical analysis was performed using the paired Student’s t test.

### Experimental murine malaria infection

*P. yoelii 17XNL GP66* and *P. yoelii 17XL* were maintained by passage through donor mice with no more than 3 inoculations prior to recirculation through mosquitoes. Infections were induced by intraperitoneal injection of 10^6^ infected erythrocytes from donor mice with parasitemia of 1–5%. The transgenic parasite stably expressing the GP66 epitope was generated as previously described [24].

### Tetramer production

Biotinylated I-A^b^ LCMV GP 66–77 DIYKGVYQFKSV monomers were obtained from the NIH tetramer core and tetramerized with SA-APC as previously described [95]. For antigen-specific B cell experiments, a 14 kDA truncated carboxy terminus of *Py*MSP1 was cloned, purified, biotinylated, and tetramerized with streptavidin-PE (Prozyme) [12,96]. Decoy reagent to detect B cells specific for tetramer components was constructed as previously described [58,97].

### Cell enrichment, flow cytometry, and antibodies

Single cell suspensions of spleen and cervical, mediastinal, axillary, brachial, pancreatic, renal, mesenteric, inguinal and lumbar lymph nodes (SLO) were prepared by mashing through Nitex mesh (amazon.com) and resuspending in 2% FBS and Fc Block (2.4G2). Cells were then stained with decoy reagent at a concentration of 10 nM at room temperature for 15 minutes, followed by MSP1-PE tetramer for 30 minutes on ice, washed, and then stained with anti-PE beads prior to a magnetic enrichment. All bound cells then were stained with antibodies shown in Supplemental Table 1, detected on an LSRII Flow Cytometer (BD Biosciences), and analyzed using Flowjo 9.94 (Treestar).

### ELISA-based malaria-specific antibody assay

96 well ELISPOT plates (Millipore) were coated overnight at 4 C with MSP1+ protein at 1 µg/mL. Plates were then blocked with 5% dehydrated milk prior to sample incubation. Plates were incubated with serially diluted serum. Bound antibodies were detected using either IgM Biotin (Clone II/41) or IgG_2_ _c_ Biotin (Clone 5.7) followed by Streptavidin-HRP (BD). Absorbance was measured at 450 nm using an iMark Microplate Reader (Bio-Rad).

## Statistics

When data were parametric, unpaired, two-tailed Student’s t tests were applied to determine the statistical significance of the difference between groups with Prism 6 (Graphpad) software.

## Supplementary Material

Supplemental Material
